# Two Atypical Cases of Hantavirus Infection: Experience from a Tertiary Care Unit in Sri Lanka

**DOI:** 10.1155/2021/5555613

**Published:** 2021-04-21

**Authors:** S. Rupasinghe, S. Bowattage, L. Herath, A. Rajaratnam

**Affiliations:** National Hospital-Kandy, Kandy, Sri Lanka

## Abstract

Hantaviruses are a large family of enveloped viruses with two medically important families Cricetidae and Muridae which are known to cause rodent-borne diseases worldwide. Some strains cause clinical syndromes with multiorgan involvement in humans such as hantavirus haemorrhagic fever with renal syndrome (HFRS) and hantavirus cardiopulmonary syndrome (HCPS), which is also known as hantavirus pulmonary syndrome. Clinical differentiation of this infection from other endemic infections in Sri Lanka such as leptospirosis and rickettsial infections is extremely difficult due to overlapping clinical and epidemiologic features such as exposure to rodents and farming. Here, we report two serologically confirmed cases of hantavirus infection from Sri Lanka with different presentation. The first patient had a combination of HCPS and HFRS. The second patient was treated for HPS complicated with acute respiratory distress syndrome (ARDS). Both had a significant clinical, biochemical, and radiological response with early initiation of corticosteroids. However, further studies are required to assess whether steroids hasten the recovery of severe hantavirus infections. We believe that hantavirus infection is an important emerging disease in the country and should be considered as a differential diagnosis in patients presenting with an acute febrile illness as well as in patients presenting with ARDS. Early diagnosis and prompt treatment improve prognosis.

## 1. Introduction

Hantaviruses are single-stranded ribonucleic acid viruses that belong to the family Bunyaviridae. Transmission to human occurs through direct contact with urine, faeces, and saliva of infected rodents and by inhalation of contaminated aerosols. Hantaviruses cause asymptomatic infections in their natural hosts, in which lifelong shedding of virus in excreta occurs. Different types of hantaviruses cause acute febrile diseases in human. Hantavirus cardiopulmonary syndrome and haemorrhagic fever with renal syndrome are caused by new world and old world hantaviruses, respectively [1]. In HCPS, inhaled infectious virus is deposited in alveoli and terminal bronchioles. Activated macrophages and cytokines contribute to the onset of respiratory symptoms. In HFRS, the main site of cytokine expression is the distal nephron causing tubular dysfunction. Also, increased glomerular permeability results in massive proteinuria [2]. The disease typically follows an incubation period of two to four weeks. Serological tests are the preferred methods of diagnosis. Treatment is mainly supportive [1].

## 2. Case 1

A 30-year-old previously healthy Sinhalese male air force officer from Dambulla (Central Province, Sri Lanka) presented initially to a primary care hospital with fever for 4 days along with arthralgia, myalgia, and anorexia followed by nausea, vomiting, watery diarrhea, and a reduction in urine output from day 2 of illness.

**Figure 1:**
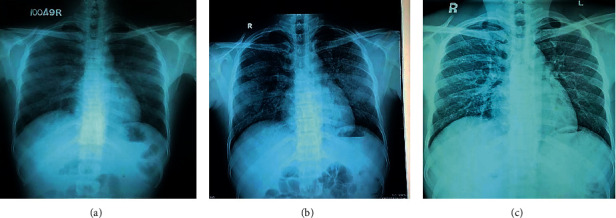
(a) CXR-PA taken on day 4 of illness (normal), (b) day 5 of illness (showing patchy opacities in peripheries of both lung fields), and (c) day 7 of illness (normal).

On physical examination at a local hospital, he had a temperature of 39.5°C, was not icteric with normal vitals (pulse rate: 96 bpm; blood pressure: 120/80 mmHg). His oxygen saturation (SpO_2_) on air was 98%. The lungs were clear, and his abdomen was soft and nontender. Since he gave a recent history of bathing in a stream and farming, he was managed as for leptospirosis with possible acute kidney injury with intravenous (IV) ceftriaxone and oral doxycycline therapy. Chest radiograph posteroanterior (CXR-PA) was normal (Figure 1(a)).

During the following day at a local hospital, on day 5 of illness, he developed nonproductive cough and shortness of breath. He was tachypneic with a drop in SpO_2_ on air to 81%. Auscultation revealed bilateral fine crepitations involving all lung zones. Repeat CXR-PA revealed bilateral peripheral alveolar opacifications (Figure 1(b)). Pulmonary hemorrhage was suspected, and he was started on dexamethasone 2 mg 8 hourly, as per local guidelines of leptospirosis management. Within 24 hours of commencement of dexamethasone therapy, his cough, dyspnea, and oxygen saturation improved. His urine output remained static at 0.5 ml/kg/hr. Since there were no facilities for haemodialysis, plasma exchange, and mechanical ventilation at the local hospital, on the 6th day of illness, he was transferred to our tertiary care center for further observation, monitoring, and management.

On admission to our unit, he denied fever but complained of a mild cough and dyspnoea. Urine output was 0.5 ml/kg/hr. He had a temperature of 37°C. His vitals were stable. However, his SpO_2_ on air was 92%, and lung auscultation revealed widespread bilateral crepitations. We continued the same IV antibiotics for a total of 10 days and promptly initiated a steroid cover with IV methyprednisolone 1 g daily for 2 days. He showed a marked improvement in cough, rise of SpO_2_ to 98%, and clear lungs by day 7 of illness. Complete chest radiographic resolution was noted on the same day (Figure 1(c)). His inflammatory markers showed improvement as shown in Table 1.

Urine full report, blood and urine cultures, dengue NS1 antigen on day 4, dengue IgM on day 6, COVID-19 PCR on day 7, and leptospira IgM and IgG performed on day 7 were negative. Electrocardiogram (ECG), 2D echocardiogram, and ultrasound of abdomen and kidneys were normal. An ELISA test for hantavirus IgM done on day 7 was positive on 1 : 100 dilutions, and repeat serum IgM against hantavirus on day 20 was also positive on 1 : 100 and 1 : 500 dilutions.

## 3. Case 2

A 37-year-old previously well male Sinhalese farmer was transferred from a primary care hospital, Ududumbara (Central Province, Sri Lanka). He presented with fever for 3 days associated with diarrhea, vomiting, nonproductive cough, and shortness of breath.

At the Emergency Treatment Unit of National Hospital Kandy, he was found to have a temperature of 40°C and was tachypneic with a respiratory rate of 36 breaths per minute and SpO_2_ of 88% on room air. Pulse rate was 104 bpm, and blood pressure was 110/70 mmHg. Lung auscultation revealed bilateral widespread fine crepitations. Abdomen was soft and nontender. Bedside arterial blood gas (ABG) revealed a pH of 7.44, PCO_2_ of 37 mmHg, PO_2_ of 71 mmHg, bicarbonate of 26 mmol/L, and PaO_2_/FiO_2_ of 119 mmHg.

Since he presented during the COVID-19 pandemic, a working diagnosis of COVID-19 or community-acquired pneumonia complicated with ARDS was made and subsequently managed in isolation at an intensive care unit (ICU) with IV ceftriaxone 1 g bd (twice daily) and IV clarithromycin 500 mg 6 hourly, with prone position ventilation via high flow nasal cannula. He was immediately commenced on IV methyl prednisolone 1 g daily which was continued for 3 days. Within 24 hours of treatment, his general condition improved. Respiratory rate came down to 20 breaths per minutes with a saturation of 98% on air with no added lung sounds on auscultation.

Improvement of laboratory markers during ICU stay is shown in Table 2.

**Figure 2:**
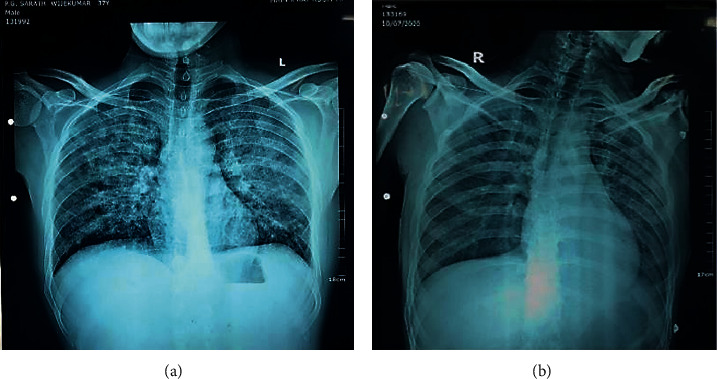
(a) CXR-PA taken on day 3 of illness (showing widespread opacities in both lung fields) and (b) on day 5 of illness (normal).

Chest radiograph prior to starting methyl prednisolone revealed bilateral extensive opacifications suggestive of ARDS (Figure 2(a)). Within 24 hours of steroid treatment, complete radiographic resolution was seen (Figure 2(b)). Hantavirus IgM done on day 3 illness was positive on 1 : 100 dilutions and on day 25 was positive on 1 : 100 and 1 : 500 dilutions. Dengue NS1 antigen test done on day 3 illness was negative. Dengue IgM and leptospirosis IgM done on day 6 illness were negative. ECG, troponin I, and 2D echo were normal. Serum procalcitonin was negative. Antibiotics were withheld on day 3 of illness. On day 5 of illness, he was asymptomatic with a SpO_2_ of 99% on air. Lungs were clear. ABG was normal with a normal PaO_2_/FiO_2_ ratio.

## 4. Discussion

Hantaviruses are zoonotic viruses which cause HCPS in America and HFRS in other parts of the world, mainly in East Asia (Korea, China, and Russia) and Europe. HCPS and HFRS are both immunopathologic [2]. HCPS begins with a prodromal phase of 3-4 days, of nonspecific symptoms including nausea, vomiting, dizziness, and cough. The prodrome is followed by pulmonary oedema, hypoxaemia, tachycardia, and hypotension. Severe infection can give rise to metabolic acidosis. Orthostatic hypotension can progress into cardiogenic shock. Overall mortality of pulmonary syndrome is 50% to 70% [3]. HFRS has five phases such as febrile, hypotensive, oliguric, polyuric, and convalescent. Fever is usually present for 3–7 days followed by conjunctival haemorrhages, palatal petechial, and hypotension. Severe haemorrhagic disease can present as epistaxis, haematamesis, haematuria, melaena, and fatal intracranial haemorrhages. Oliguria typically lasts from 3 to 7 days with a transient decrease in renal function. This is followed by the polyuric phase with improvement of renal functions and full recovery over next 6 months without significant complications [3].

The patient in our first case had an overlap of both HFRS and HCPS. There are few similar case reports in available literature as follows. Typically, HFRS occurs in Asia and Europe, and HCPS is commonly seen in North and South America. It is special that these cases occurred in an island nation in Asia. It is also interesting that two similar cases have been reported from nearby areas in the past. Those cases also were initially misdiagnosed as leptospirosis.

The patient in our second case was diagnosed as having HCPS complicated with ARDS. Even though ARDS has been described as a complication of HCPS, case reports of hantavirus infection with ARDS are rare in available literature. Murthy et al. from India described a case of HPS with ARDS and multiorgan dysfunction in a postpartum woman, in 2016 [4].

Chest X-ray in HCPS may reveal bilateral pulmonary oedema in initial days, in one-third of patients. Two-thirds of patients may develop bibasilar opacities and pleural effusions [2]. Both of our cases had bilateral opacifications. Ketai et al. reported that HCPS can be differentiated from ARDS by chest radiography. According to that, HCPS may show a normal chest X-ray during early disease. This is followed by interstitial edema, Kerley B lines, peribronchial cuffing, and indistinct hila. Over the subsequent 48 hours, progression to pulmonary oedema occurs which is indicated by centrally located dense alveolar infiltrates unlike the more peripheral infiltrates seen in ARDS. Hence, chest X-ray per se may help in early suspicion of HCPS in settings where serological marker testing is not readily available. The mean specificity and sensitivity of chest radiograph interpretation for HPS were 86 ± 13% and 74 ± 11%, respectively. Accuracy is improved by highly trained readers and using serial radiographs [5].

Other laboratory findings include thrombocytopenia, atypical lymphocytes, elevated haematocrit, hyponatraemia, a decrease in serum protein, and microscopic haematuria [2]. Diagnosis is confirmed by immunoblot assay and immunofluorescence assay (IFA). ELISA is preferred with IgM [2]. We used IFA to detect immunoglobulin M against hantavirus. In both our cases, serology was obtained during hospital stay.

Both of our cases had varied presentation, one mild and other in severe form, but had marked clinical and radiographic resolution with steroids in addition to supportive care. The place for steroids in hantavirus infection was tested in a double-blind randomized case control study which failed to demonstrate a significant clinical benefit to patients, even though it appeared to be safe [6]. But both of our patients had clinical, biochemical, and radiological improvement with steroids. In case 1 of reference 9, despite of giving methyl prednisolone, it has taken longer time to improve. The case in reference 10 may have recovered soon due to the effect of methyl prednisolone. However, large randomized trials are needed to assess the efficacy of steroids in hantavirus infection. The place for ribavirin in hantavirus infection is controversial [7]. However, ribavirin was not used in our cases due to unavailability of the drug.

Hantavirus was first described in Sri Lanka by Vitarana et al. in 1988 by demonstrating positive serology in four patients out of 248 patients who presented with symptoms similar to leptospirosis [8]. Gamage et al., in 2011, reported 8 cases of hantavirus infection during an outbreak of leptospirosis, at Peradeniya Hospital, Sri Lanka [9]. In 2018, Ehelepola et al. described two atypical cases of hantavirus presenting with a combination of pulmonary and renal symptoms similar to the patient in our first case [10]. Recently in 2020, Dalugama et al. reported an atypical case of hantavirus mimicking leptospirosis [11]. We believe that hantavirus infections are infrequently reported from Sri Lanka and other Asian countries due to nonavailability of diagnostic facilities and since it is being clinically misdiagnosed as leptospirosis. As our second case, cases of hantavirus complicated with ARDS are rare in available literature.

## 5. Conclusion

We believe that hantavirus infection is an important emerging zoonosis and should be considered as a differential diagnosis of acute febrile illness along with leptospirosis, dengue fever, and scrub typhus. It should also be considered in patients presenting with ARDS. Typical chest radiograph findings of HCPS may support the diagnosis in settings without serological testing facilities. Early initiation of steroids may have contributed to hasten recovery and shorten hospital stay of these two patients. However, large randomized trials are needed to assess the efficacy of steroids in hantavirus infection.

## Figures and Tables

**Table 1:** Laboratory investigations.

	Day 4 illness	Day 7 illness	Day 10 illness
White cell count	20 × 10^9^/L	13 × 10^9^/L	12.32 × 10^9^/L
Platelet count	101 × 10^9^/L	162 × 10^9^/L	391 × 10^9^/L
Hemoglobin	13.5 g/dL	13.6 g/dL	13 g/dL
Haematocrit	41.3%	42.1%	40.8%
Aspartate transaminase (AST)	102 U/L	78 U/L	70 U/L
Alanine transaminase (ALT)	78 U/L	65 U/L	62 U/L
Serum creatinine	0.8 mg/dL	1.34 mg/dL	0.7 mg/dL
Erythrocyte sedimentation rate	31 mm/hr	—	—
C-reactive protein (CRP)	29 mg/L	26.5 mg/L	25 mg/L

**Table 2:** Laboratory investigations.

	Day 3 of illness	Day 6 of illness	Day 8 of illness
White cell count	11.07 × 10^9^/L	9.41 × 10^9^/L	8.10 × 10^9^/L
Platelet count	165 × 10^9^/L	209 × 10^9^/L	258 × 10^9^/L
Hemoglobin	13.5 g/dL	13.3 g/dL	13.1 g/dL
Haematocrit	45%	44.3%	44.1%
Creatinine	0.78 mg/dL	0.8 mg/dL	0.8 mg/dL
AST	69 U/L	103 U/L	77 U/L
ALT	64 U/L	77.7 U/L	73 U/L
CRP	3.2 mg/L	3.2 mg/L	3 mg/L
